# Etiology of Acute Lower Respiratory Illness Hospitalizations Among Infants in 4 Countries

**DOI:** 10.1093/ofid/ofad580

**Published:** 2023-11-16

**Authors:** John Kubale, Stephanie Kujawski, Irena Chen, Zhenke Wu, Ilham Abu Khader, Iris Hasibra, Brett Whitaker, Lionel Gresh, Artan Simaku, Eric A F Simões, Mahmoud Al-Gazo, Shannon Rogers, Susan I Gerber, Angel Balmaseda, Veronica L Tallo, Tareq M Al-Sanouri, Rachael Porter, Silvia Bino, Eduardo Azziz-Baumgartner, Meredith McMorrow, Danielle Hunt, Mark Thompson, Holly M Biggs, Aubree Gordon

**Affiliations:** Institute for Social Research, University of Michigan, Ann Arbor, Michigan, USA; Epidemic Intelligence Service, US Centers for Disease Control and Prevention, Atlanta, Georgia, USA; National Center for Immunization and Respiratory Diseases, US Centers for Disease Control and Prevention, Atlanta, Georgia, USA; Department of Biostatistics, School of Public Health, University of Michigan, Ann Arbor, Michigan, USA; Department of Biostatistics, School of Public Health, University of Michigan, Ann Arbor, Michigan, USA; The Eastern Mediterranean Public Health Network, Amman, Jordan; Department of Epidemiology and Control of Infectious Diseases, Institute of Public Health, Tirana, Albania; National Center for Immunization and Respiratory Diseases, US Centers for Disease Control and Prevention, Atlanta, Georgia, USA; Sustainable Sciences Institute, Managua, Nicaragua; Department of Epidemiology and Control of Infectious Diseases, Institute of Public Health, Tirana, Albania; Section of Infectious Diseases, Department of Pediatrics, University of Colorado School of Medicine, Aurora, Colorado, USA; Center for Global Health, Department of Epidemiology, Colorado School of Public Health, Aurora, Colorado, USA; The Eastern Mediterranean Public Health Network, Amman, Jordan; National Center for Immunization and Respiratory Diseases, US Centers for Disease Control and Prevention, Atlanta, Georgia, USA; National Center for Immunization and Respiratory Diseases, US Centers for Disease Control and Prevention, Atlanta, Georgia, USA; Sustainable Sciences Institute, Managua, Nicaragua; Centro Nacional de Diagnóstico y Referencia, Ministry of Health, Managua, Nicaragua; Department of Health, Research Institute for Tropical Medicine, Muntinlupa City, Metro Manila, Philippines; The Eastern Mediterranean Public Health Network, Amman, Jordan; National Center for Immunization and Respiratory Diseases, US Centers for Disease Control and Prevention, Atlanta, Georgia, USA; Department of Epidemiology and Control of Infectious Diseases, Institute of Public Health, Tirana, Albania; National Center for Immunization and Respiratory Diseases, US Centers for Disease Control and Prevention, Atlanta, Georgia, USA; National Center for Immunization and Respiratory Diseases, US Centers for Disease Control and Prevention, Atlanta, Georgia, USA; Abt Associates, Inc, Atlanta, Georgia, USA; National Center for Immunization and Respiratory Diseases, US Centers for Disease Control and Prevention, Atlanta, Georgia, USA; National Center for Immunization and Respiratory Diseases, US Centers for Disease Control and Prevention, Atlanta, Georgia, USA; Department of Epidemiology, School of Public Health, University of Michigan, Ann Arbor, Michigan, USA

**Keywords:** etiology, global health, influenza virus, respiratory infections, RSV

## Abstract

**Background:**

Recent studies explored which pathogens drive the global burden of pneumonia hospitalizations among young children. However, the etiology of broader acute lower respiratory tract infections (ALRIs) remains unclear.

**Methods:**

Using a multicountry study (Albania, Jordan, Nicaragua, and the Philippines) of hospitalized infants and non-ill community controls between 2015 and 2017, we assessed the prevalence and severity of viral infections and coinfections. We also estimated the proportion of ALRI hospitalizations caused by 21 respiratory pathogens identified via multiplex real-time reverse transcription polymerase chain reaction with bayesian nested partially latent class models.

**Results:**

An overall 3632 hospitalized infants and 1068 non-ill community controls participated in the study and had specimens tested. Among hospitalized infants, 1743 (48.0%) met the ALRI case definition for the etiology analysis. After accounting for the prevalence in non-ill controls, respiratory syncytial virus (RSV) was responsible for the largest proportion of ALRI hospitalizations, although the magnitude varied across sites—ranging from 65.2% (95% credible interval, 46.3%–79.6%) in Albania to 34.9% (95% credible interval, 20.0%–49.0%) in the Philippines. While the fraction of ALRI hospitalizations caused by RSV decreased as age increased, it remained the greatest driver. After RSV, rhinovirus/enterovirus (range, 13.4%–27.1%) and human metapneumovirus (range, 6.3%–12.0%) were the next-highest contributors to ALRI hospitalizations.

**Conclusions:**

We observed substantial numbers of ALRI hospitalizations, with RSV as the largest source, particularly in infants aged <3 months. This underscores the potential for vaccines and long-lasting monoclonal antibodies on the horizon to reduce the burden of ALRI in infants worldwide.

Acute lower respiratory tract infection (ALRI), such as pneumonia and bronchiolitis, is a leading cause of morbidity and mortality in children aged <5 years [1–4]. Infants aged <1 year are disproportionately affected and account for a high burden of hospital admissions for lower respiratory tract infections [2]. Common causes of viral respiratory infections in children include respiratory syncytial virus (RSV), rhinovirus, and influenza [4]. Previous studies have assessed the etiology for pneumonia among children aged <5 years (ie, Pneumonia Etiology Research for Child Health [PERCH]) [5], but important gaps remain in the literature.

Particularly important among these gaps is an understanding of the etiology of ALRI among the youngest and most vulnerable children: those aged <1 year. Data from diverse settings are also vital to account for the substantial geographic variation that exists in disease transmission (eg, PERCH centered on Africa and Asia). Finally, multiplex laboratory methods that test for several respiratory pathogens at once, when combined with study designs that include controls, allow for a better understanding of respiratory pathogen prevalence and causes of respiratory infections. However, studies based on multiplex detection methods have frequently relied on testing criteria that may limit their sensitivity (eg, fever) [6–8]. Testing all infants, regardless of symptom presentation, allows for a more comprehensive understanding of viral pathogen prevalence and the associated clinical manifestations. Finally, reliance on chest radiographs for pneumonia diagnosis (as in PERCH) is not always possible, particularly in resource-limited settings; even when used as part of pneumonia diagnosis, such radiographs offer inferior sensitivity and specificity [9]. Exploring the viral etiology of hospitalized ALRI without relying on chest radiographs could allow for a broader understanding of lower respiratory infection in children.

In this study, we aimed to determine the prevalence and epidemiologic characteristics of 21 respiratory pathogens and their role in driving ALRI hospitalizations within a cohort of hospitalized infants (aged <1 year) and non-ill community controls in 4 middle-income countries: Albania, Jordan, Nicaragua, and the Philippines.

## METHODS

### Study Design and Participants

**Figure 1.**
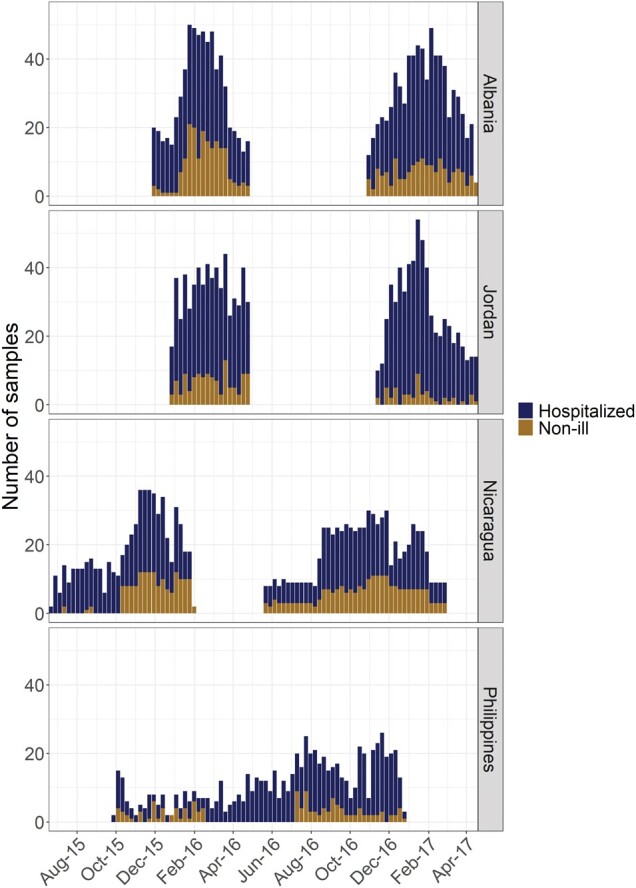
Hospitalized and non-ill participant enrollment (by number of samples per week) over time by study site. The dark blue bars reflect the number of enrollment samples from hospitalized infants at each site, while the brown bars show the number taken from non-ill community controls.

The Influenza and Respiratory Syncytial Virus in Infants Study used previously described methods [10–12]. Briefly, 1 hospital from each of the following countries was included: Albania, Jordan, Nicaragua, and the Philippines. Infants aged <1 year who were hospitalized for an acute illness at these hospitals were enrolled over 2 consecutive years (2015–2016 and 2016–2017) in Albania, Jordan, and Nicaragua (Figure 1). In year 1, the start of enrollment coincided with influenza circulation, while in year 2 it also coincided with RSV circulation. In the Philippines, enrollment occurred during 1 continuous 34-week period from September 2015 to December 2016. This timing was chosen because prior evidence at the study sites showed that few ALRI hospitalizations occurred outside of these seasons. Because of this, the study was not designed to conduct surveillance during those periods. To be eligible for the study, hospitalized infants needed to be admitted within 24 hours, reside within the hospital catchment area, and be admitted within 10 days of illness onset [10].

A community cohort of non-ill infants was enrolled concurrent with the hospitalized cohort during immunization visits. In addition, non-ill children in Jordan were enrolled during routine preventive care visits, while non-ill infants in Nicaragua were enrolled from an existing cohort study [10, 13, 14]. Non-ill infants were eligible for enrollment if parents reported no acute illness within the past 7 days. Weekly enrollment goals were set stratified by age (0–5 and 6–11 months) to reflect the distribution of hospitalized infants.

### Data Collection

For the hospitalized cohort, the following data were collected from parent interviews and medical chart abstraction: demographics (eg, age, sex, education, smoking status, breastfeeding status, premature birth, low birth weight, infant health status) and clinical information (symptoms, underlying medical conditions, highest level of care, vital signs, clinical interventions, discharge diagnosis). Respiratory specimens (combined nasal-oropharyngeal swabs) were acquired within 24 hours of hospital admission. For the non-ill cohort, respiratory specimens were collected during enrollment. In the second year of the study, parents of a sample of non-ill infants were interviewed 4 to 10 days after enrollment to determine whether their children had developed symptoms.

### Laboratory Methods

Respiratory specimens were tested by multiplex real-time reverse transcription polymerase chain reaction (RT-PCR; FTD Respiratory Pathogens 21 [15]) for the following pathogens: rhinoviruses/enteroviruses (RV/EV), seasonal coronaviruses (NL63, 229E, OC43, HKU1), human parainfluenza virus (HPIV) 1 to 4, human metapneumovirus (HMPV) A and B, human bocavirus, human adenovirus (HAdV), *Mycoplasma pneumoniae*, parechoviruses, RSV, and influenza A and B. Positive RT-PCR results were considered indicative of infection or coinfection. Most specimens were tested at study site laboratories, though a subset from Jordan and Albania was tested at the US Centers for Disease Control and Prevention (CDC). Prior to testing, all laboratories demonstrated proficiency with the multiplex panel identifying pathogens from blinded samples distributed by the CDC. For influenza and RSV, respiratory specimens were tested with singleplex assays provided by the CDC. Detailed laboratory methods for influenza and RSV are published elsewhere [11, 16].

### Statistical Analysis

We calculated the prevalence of infections and coinfections for the entire cohort (number of PCR^+^/total number tested), stratified by study site, age (<3, 3–5, and 6–11 months), and sex. We also assessed the relative severity of single infections vs coinfections and examined the etiology of hospitalized ALRI. All analyses were performed with R version 4.2.1.

### Severity of Single vs Coinfections

We characterized the severity of single vs coinfections among hospitalized infants by fitting negative binomial models with days hospitalized serving as a proxy measure for illness severity. We were powered to assess potential differences in severity only between RV/EV + RSV and RV/EV + HAdV coinfections, the 2 most frequent combinations (Supplementary Figure 1). We adjusted for age, sex, and comorbid conditions in both models.

### Etiology of ALRI

To assess the proportion of ALRI hospitalizations in the study period caused by a range of respiratory pathogens (population etiologic fraction), we excluded those in the hospitalized cohort without ALRI and fit bayesian nested partially latent class models using the *baker* package in R [17, 18]. This modeling approach was used in the PERCH study to characterize the etiology of pneumonia hospitalizations among children aged <5 years [5]. We expanded on this research by including ALRI cases defined by pneumonia, acute respiratory distress syndrome, bronchiolitis, or respiratory failure, regardless of chest radiograph availability. Our models estimated the proportion of ALRI hospitalizations caused by different respiratory pathogens. The models treated the true pathogens as unknown variables (“latent variables”) and used control data to estimate detection rates [18–20]. In a bayesian framework, the *baker* package implements a posterior inference algorithm to estimate the fractions of cases caused by each causative pathogen with the uncertainty quantification of the estimated fractions. The models considered potential correlations among multiple pathogens and included an “other/unknown” class to account for untested pathogens. The models assumed that a single pathogen caused each ALRI hospitalization, even if multiple pathogens were detected in the same sample. The population etiologic fraction for a pathogen, however, can be interpreted (loosely) as the contribution by said pathogen to ALRI hospitalizations, be it from a singleton or nonsingleton cause. We reported estimated posterior means and 95% credible intervals (CrIs) for model predictors. Additional information—such as sensitivity analyses (Supplementary Figure 2) and detail on the nested partially latent class model, including prior specification—is available in the supplemental materials.

### Patient Consent Statement

The study protocol was approved by the institutional review board at each study site. Parents provided written informed consent for their children to participate in the study.

## RESULTS

A total of 4969 infants participated in the study, with 3634 enrolled into the hospitalized cohort and 1335 enrolled as non-ill community controls. Respiratory samples were available for testing from 3632 hospitalized participants and 1068 non-ill controls. Overall, 56.7% of participants were male, though this varied by site and cohort (range, 46.1%–63.9%). Similarly, 14.4% of participants were classified as low birthweight (<2500 g), though the proportion again varied by site and cohort (range, 4.2%–24.0%; Table 1).

**Table 1.** Demographics of Participants by Site and Cohort

	Albania		Jordan		Nicaragua		Philippines	
Characteristic	Hospitalized (n = 1032)	Non-ill (n = 363)	Hospitalized (n = 1056)	Non-ill (n = 173)	Hospitalized (n = 936)	Non-ill (n = 399)	Hospitalized (n = 607)	Non-ill (n = 133)
ALRI	585 (57)	…	414 (39)	…	427 (46)	…	317 (52)	…
Age, wk^a^	20.54 (15.00)	22.20 (14.21)	11.34 (12.64)	23.63 (16.20)	20.61 (16.19)	19.91 (14.86)	22.06 (16.24)	15.14 (14.41)
Male	586 (57)	211 (58)	602 (57)	86 (50)	534 (57)	184 (46)	375 (62)	85 (64)
Parent education^b^	717 (70)	296 (82)	678 (64)	115 (66)	483 (58)	200 (56)	458 (76)	122 (92)
Low birthweight	90 (8.9)	15 (4.2)	246 (24)	20 (12)	89 (10)	25 (7.0)	132 (23)	27 (21)
Smoking in household								
Never	603 (59)	309 (85)	280 (27)	49 (28)	572 (61)	279 (71)	247 (41)	75 (56)
<Monthly	35 (3.4)	0 (0)	1 (<0.1)	1 (0.6)	7 (0.7)	5 (1.3)	0 (0)	0 (0)
Monthly	19 (1.8)	2 (0.6)	9 (0.9)	0 (0)	13 (1.4)	0 (0)	1 (0.2)	0 (0)
Weekly	61 (5.9)	8 (2.2)	31 (2.9)	5 (2.9)	62 (6.6)	22 (5.6)	19 (3.1)	5 (3.8)
Daily	311 (30)	43 (12)	734 (70)	118 (68)	280 (30)	89 (23)	337 (56)	53 (40)

Data are presented as No. (%) unless noted otherwise.

Abbreviation: ALRI, acute lower respiratory tract infection.

^a^Mean (SD).

^b^Secondary or higher.

### Prevalence

#### Hospitalized Cohort.

**Figure 2.**
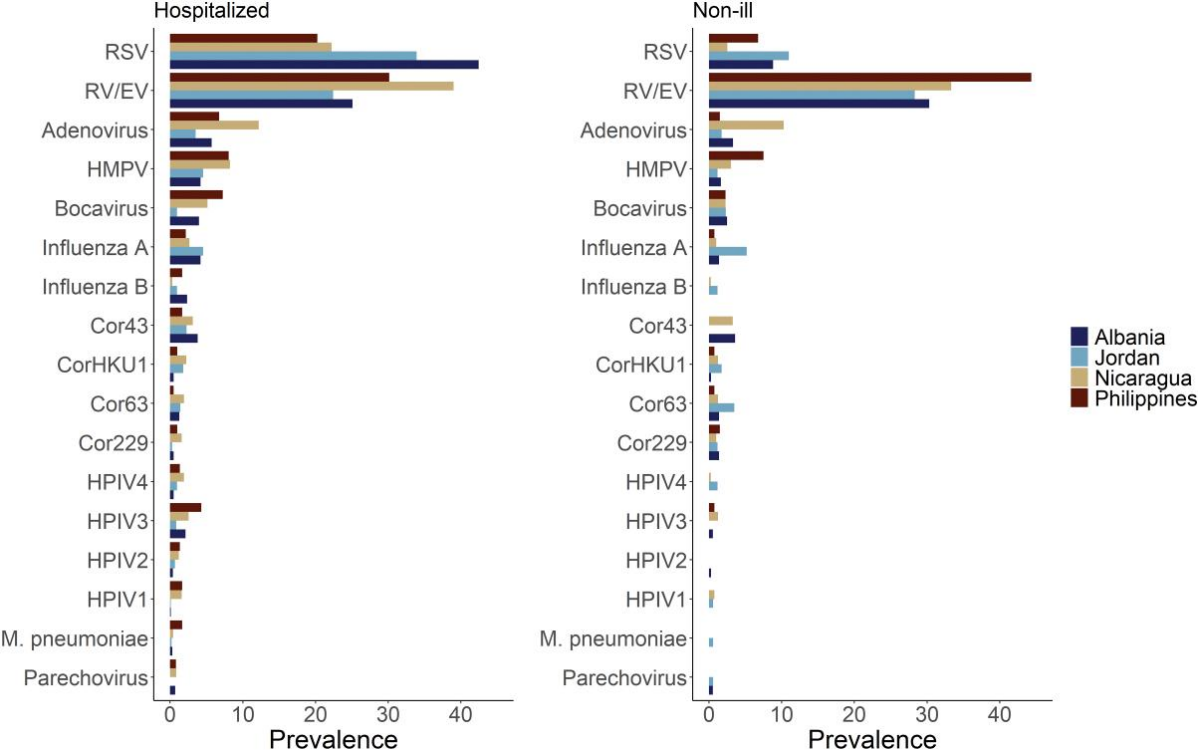
Viral prevalence by site and cohort. *Left,* The prevalence of all pathogens by site among hospitalized participants: number positive for each pathogen/number tested, stratified by study site and cohort (hospitalized or non-ill). *Right*, The prevalence of all pathogens by site among non-ill community controls. Cor229, coronavirus 229E; Cor43, coronavirus OC43; Cor63, coronavirus NL63; CorHKU1, coronavirus HKU1; HMPV, human metapneumovirus; HPIV1-HPIV4, human parainfluenza virus 1–4; RSV, respiratory syncytial virus; RV/EV, rhinovirus/enterovirus.

At least 1 pathogen was detected in 2547 (70.1%) of the 3632 hospitalized infants (Figure 2, Supplementary Tables 1 and 2). The most common infections were RSV (31.1%) and RV/EV (28.7%), followed by HAdV (6.9%), coronaviruses (6.2%), HMPV (6.0%), HPIV (4.9%), and influenza (4.9%). Approximately a quarter of infants with an infection (n = 709, 27.8%) had >1 pathogen detected and were classified as coinfections. Among those with coinfections, the most common combinations had RV/EV (67.6%) and RSV (48.7%) as an infecting pathogen (Supplementary Figure 1). The prevalence of infections varied by study site. RSV and RV/EV were the 2 most common infections detected, with RSV the most prevalent pathogen in Albania and Jordan and RV/EV more prevalent in Nicaragua and the Philippines. Similarly, the third- to fifth-most prevalent pathogens in Albania (adenovirus, influenza A, and HMPV) matched those of Jordan (albeit in a slightly different order), while Nicaragua and the Philippines had bocavirus rather than influenza A. Patterns of pathogen prevalence varied over time and by study site (Supplementary Figure 3).

#### Non-ill Cohort.

Almost half of non-ill participants (48.2%, n = 513) had at least 1 pathogen detected, a significantly smaller proportion (chi-square, *P* < .0001) than that observed among hospitalized participants. The most common pathogen detected was RV/EV (32.9%), followed by RSV (6.6%), coronaviruses (6.2%), and HAdV (5.4%; Supplementary Table 1). In study year 2, 526 infants with multiplex results were followed up after specimen collection to assess for the development of respiratory symptoms. Of these infants, 92 (17.5%) developed symptoms (within 4–10 days of enrollment), with most testing positive for at least 1 pathogen (n = 56, 60.9%; Supplementary Table 3). The highest percentage of respiratory symptom development occurred among those who tested positive for RSV (40.5%) and HMPV (28.6%), and the most common symptom reported was nasal congestion/runny nose.

### Coinfection Occurrence and Severity

Codetections were more common among hospitalized infants, with 722 (19.8%) testing positive for >1 pathogen as opposed to 119 (11.1%) of non-ill community controls (*P* < .001). Specifically, in the hospitalized cohort, 599 (83.0%) tested positive for 2 pathogens, 112 (15.5%) for 3, and 11 (1.5%) for 4. Among hospitalized participants, the most frequent coinfection combinations involved RSV and RV/EV, representing 28.4% (n = 205) of all coinfections (Supplementary Figure 1). Only 38 (18.5%) of these participants with RSV and RV/EV coinfections had additional pathogens detected. No difference was observed in the duration of hospitalization between single infections and coinfections in either comparison (Supplementary Figure 4).

### ALRI Etiology

**Figure 3.**
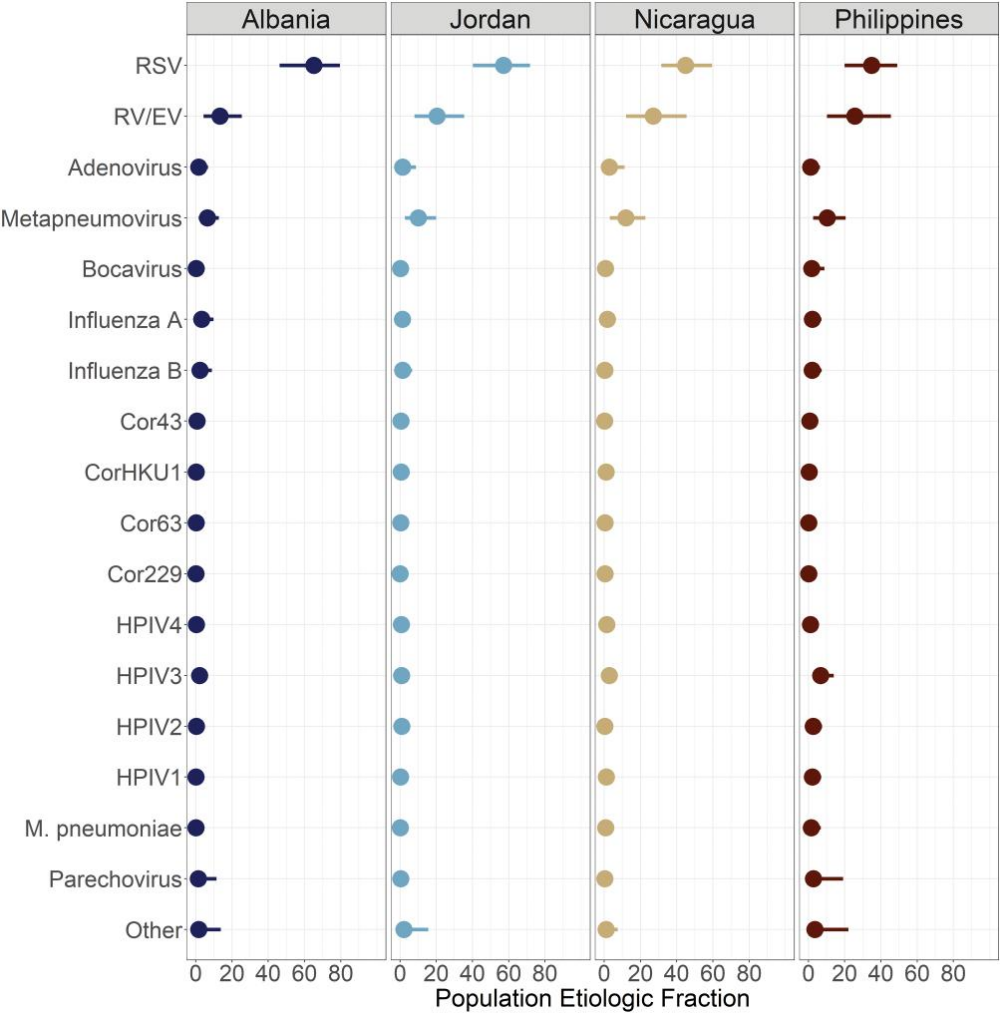
Population etiologic fraction for each pathogen by study site (marginalized over sex and age). The points represent the mean population etiologic fraction for a given pathogen from the posterior distribution, while the bar corresponds to the 95% credible interval. A pathogen's population attributable fraction can be interpreted as the proportion of ALRI hospitalizations at a site that were attributable to specific ALRI. For example, in Albania 65.2% (95% CrI, 46.3–79.6) of ALRI hospitalizations among infants were attributable to RSV. ALRI, acute lower respiratory tract infection; Cor229, coronavirus 229E; Cor43, coronavirus OC43; Cor63, coronavirus NL63; CorHKU1, coronavirus HKU1; HMPV, human metapneumovirus; HPIV1-HPIV4, human parainfluenza virus 1–4; RSV, respiratory syncytial virus; RV/EV, rhinovirus/enterovirus.

**Figure 4.**
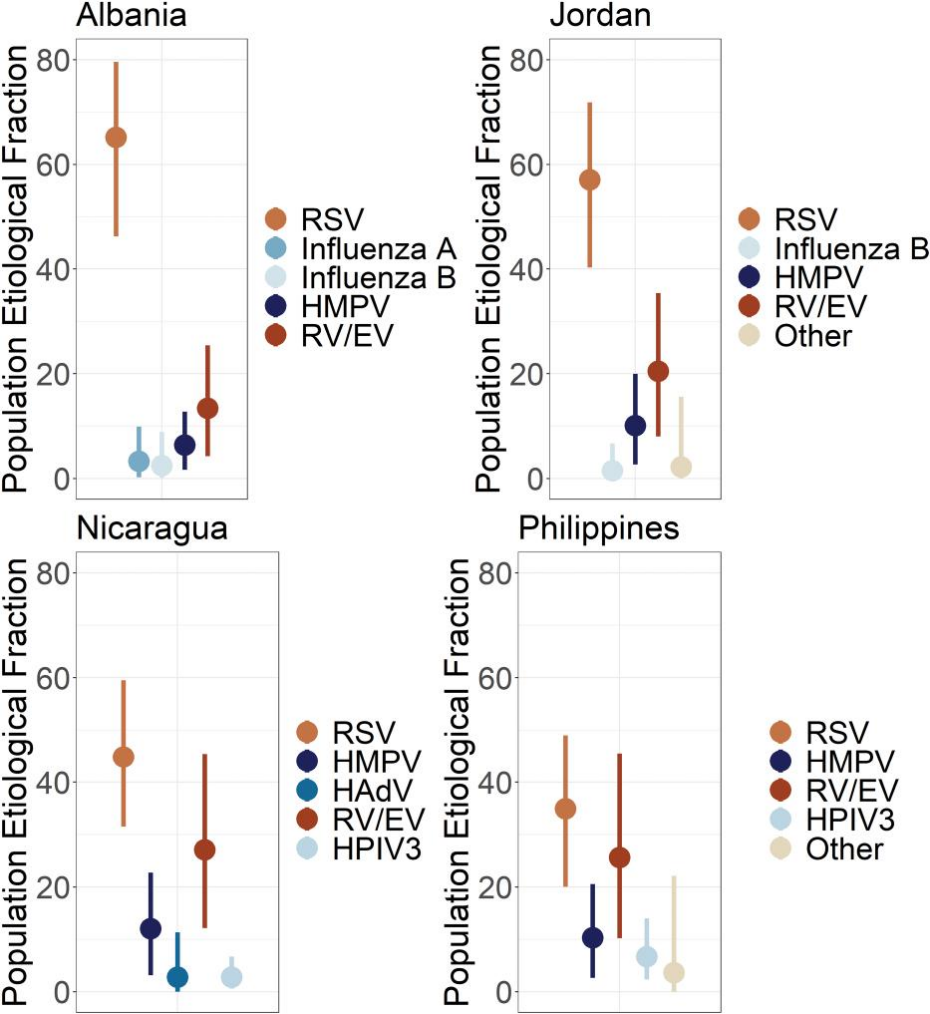
Top 5 etiologies of acute lower respiratory tract infection hospitalizations by study site. Points represent the posterior mean of the population etiologic fraction of each pathogen by site. Lines reflect the 95% credible intervals. HAdV, human adenovirus; HMPV, human metapneumovirus; HPIV3, human parainfluenza virus; RSV, respiratory syncytial virus; RV/EV, rhinovirus/enterovirus.

**Figure 5.**
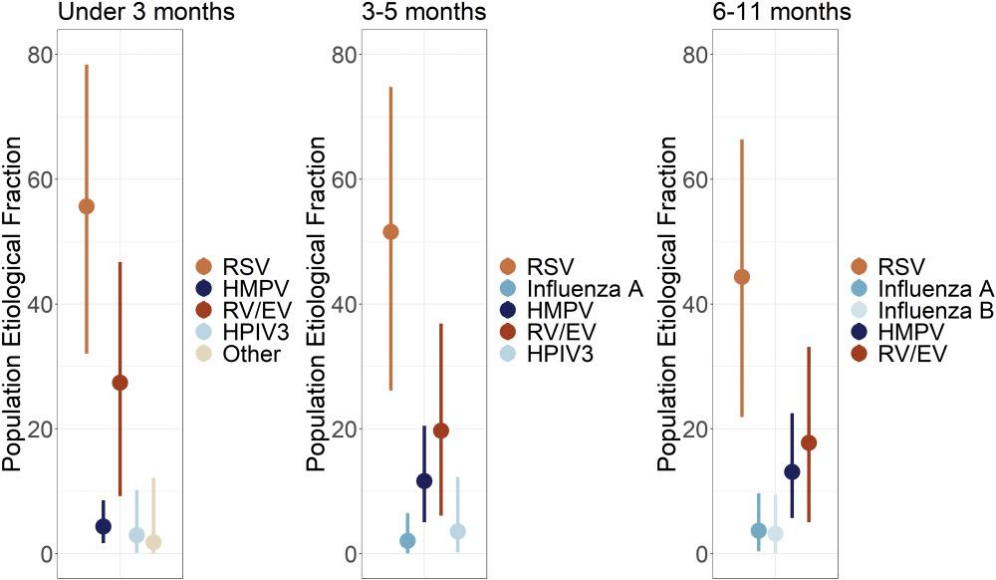
Top 5 etiologies of acute lower respiratory tract infection hospitalizations by age group. Points represent the posterior mean of the population etiologic fraction of each pathogen by site. Lines reflect the 95% credible intervals. HMPV, human metapneumovirus; HPIV3, human parainfluenza virus; RSV, respiratory syncytial virus; RV/EV, rhinovirus/enterovirus.

A total of 1743 (48.0%) hospitalized infants met the ALRI case definition for inclusion in the etiology analysis with all non-ill community controls. At each study site, a relatively small number of pathogens were responsible for the majority of ALRI hospitalizations during the study period. In fact, for Albania, Jordan, and Nicaragua, approximately 90% of ALRI hospitalizations in the study period were caused by 5 pathogens (range, 89.5%–91.5%), while in the Philippines this proportion was slightly lower at 81.2% (Figures 3 and 4). RSV was by far the largest driver of ALRI hospitalizations during the study, responsible for an estimated 31.4% (95% CrI, 28.7%–34.0%) in the population (Figure 5, Supplementary Table 4). HMPV and RV/EV were the next-largest contributors, associated with 6.6% (95% CrI, 5.0%–8.1%) and 5.9% (95% CrI, 4.2%–8.0%) of ALRI hospitalizations, respectively. While RSV, RV/EV, and HMPV were consistently observed as the top 3 causes of ALRI hospitalizations during the study period, the pathogens occupying the fourth and fifth slots differed depending on site and age (Figures 4 and 5). Additionally, although RSV was regularly the largest driver of ALRI hospitalizations across age, sex, and study site, there was variability in its magnitude with site-specific estimates ranging from 34.9% in the Philippines to 65.2% in Albania. When examined by age, RSV was typically responsible for the largest proportion of ALRI hospitalizations in the study period among children aged <3 months, with this proportion decreasing as age increased. RV/EV followed a similar pattern as RSV (decreasing with age), while HMPV showed the opposite—increasing among older infants.

## DISCUSSION

In this multicountry study among infants, we found that 70% of infants hospitalized for acute illness tested positive for at least 1 respiratory virus. The most common infections on multiplex RT-PCR were RSV, RV/EV, HAdV, coronaviruses, HMPV, and HPIV. Coinfection with RSV + RV/EV or HAdV + RV/EV did not seem to be associated with greater duration of hospitalization when compared with a single infection. RSV was by far the largest driver of ALRI hospitalizations throughout the study period regardless of age and country. There was, however, substantial variation in magnitude across these groups, with those aged <3 months and those in Albania having the greatest proportion of ALRI hospitalizations attributable to RSV. There appeared to be less variation (vs PERCH [5]) in the proportion of ALRI hospitalizations attributable to pathogens other than RSV, suggesting that RSV is a particularly important driver of ALRI burden in the first year of life.

Our estimates for the prevalence of viral infections and coinfections among hospitalized infants were largely consistent with previous studies in the literature. In Jordan, a study found that among children aged <2 years who were hospitalized with acute respiratory infection and/or fever (and tested for 11 viruses), 82% had at least 1 virus detected and 37% had a viral coinfection—with RSV, RV, HAdV, HMPV, and PIV the most frequently detected [7]. Similarly, a study in Spain found that among acute respiratory infection hospitalizations in children aged 0 to 14 years, 75% had at least 1 virus detected, with RSV (31%), RV (30%), HAdV (21%), and HMPV (6%) being most common.

Although the severity of coinfections has been studied extensively since the widespread availability of multiplex PCR testing, consensus remains elusive [21–23]. Some studies have reported increased severity of specific coinfection combinations [23], whereas others have shown no difference or even lesser severity [22]. Here we failed to detect any difference in duration of hospitalization, which we used as a proxy for illness severity. While viral coinfections may still be clinically relevant at the individual level [24], a perhaps more important question from a population health perspective is the relative importance of viral cocirculation in driving burden on the health system, as evidence suggests that viral interference may limit the frequency of coinfection [25–27].

As the PERCH analysis found with pneumonia hospitalizations among children aged <5 years, we found that RSV was the largest source of ALRI hospitalizations among infants [5]. Interestingly, though, the remaining burden of ALRI hospitalizations was more evenly distributed among the other pathogens (Figure 3). This suggests less diversity in the pathogens driving ALRI requiring hospitalization in the first year of life when compared with children aged 2 to 4 years. One area where further study is warranted is in the relative proportion of ALRI hospitalizations due to “other” causes (ie, those not included in the panel). While the proportion estimated in this study (4.5%) is greater than that reported among infants in the PERCH study (1.3%), it is perhaps not as large as we might expect given that ALRI hospitalizations caused by bacteria (not assessed in this study) would be included in this “other” proportion [5, 28]. However, this may have been associated with the enrollment criteria making viral infections more likely (ie, illness onset in prior 10 days) [29].

There were several key strengths in this study. First, 3632 hospitalized infants were enrolled and tested (1743 hospitalized infants in ALRI etiology analysis): a large sample size vital for analyses in this age group. Second, including study sites outside of Africa and Asia provides key insights into the etiology of ALRI in regions with distinct levels of burden and patterns of transmission, improving our understanding of ALRI etiology globally. Third, by focusing on infants, we were able to assess the etiology of ALRI among the population with the greatest burden, thus helping highlight priorities for reducing ALRI morbidity and mortality. Fourth, by extending the model to ALRI regardless of chest radiograph, we provided insight into settings where diagnostics are infeasible and captured additional cases that may have otherwise been excluded.

This analysis is subject to a few limitations. First, as the study took place for 2 years and participant enrollment was contingent on influenza and RSV circulation at three-quarters of the sites (Supplementary Figure 3), we were unable to characterize seasonal patterns for these viruses. Year-round data for >3 years are needed to determine seasonality [30–32]. This also means that the population etiologic fraction should be interpreted only within the study periods at each site. However, it should be noted that few ALRI hospitalizations occurred outside these periods. Second, specimens were not universally tested for bacterial pathogens (aside from *M pneumoniae*, which was part of the FTD panel), so they were excluded from this analysis. So, despite positive testing for a respiratory viral pathogen, it is possible that a nonviral and/or nonrespiratory pathogen contributed to acute illness and hospitalization. The model accounted for this with the inclusion of the “other/unknown” classification, although further study is required to assess its sensitivity. Finally, we recognize that duration of hospitalization is not an ideal measure of illness severity and may not provide a comprehensive representation of severe illnesses in the cohort. We considered using other measures (eg, ventilation, intensive care unit admission), but differences in the consistent availability of these resources across sites presented challenges for comparability.

This study shows that RSV is the most important driver of ALRI hospitalizations among children in the first year of life. This study builds on previous research, such as PERCH, by extending our understanding of ALRI etiology to additional regions, using a broader case definition, and including a greater number of infants (the age group at highest risk for severe disease). Most important, with the development of new preventatives, such as nirsevimab, and the prospect of effective vaccines on the horizon, significant reductions in infant morbidity and mortality are within reach [33, 34]. However, to achieve these reductions, it will require a commitment to delivering vaccines and monoclonal antibodies to the populations in greatest need—infants in low- and middle-income countries.

## Supplementary Material

ofad580_Supplementary_Data

## Supplementary Data

Supplementary materials are available at *Open Forum Infectious Diseases* online. Consisting of data provided by the authors to benefit the reader, the posted materials are not copyedited and are the sole responsibility of the authors, so questions or comments should be addressed to the corresponding author. R code and other information related to this analysis can be found at: https://osf.io/94753/.

## Notes

***Acknowledgements.*** We thank the participants and their families for their willingness to be a part of this study. We wish to thank other IRIS Network investigators from the CDC, Abt Associates (Rebecca Fink, Laura Edwards), Nicaragua (Felix Sanchez, Guillermina Kuan, Nery Sanchez, Sergio Ojeda, Karla Patricia Membreño), Jordan (Aktham Haddadin, Mahmoud Al-Gazo), and the Philippines (Lei Lanna Mendoza—Dancel and Karen Lana Cruz). We also thank those from the CDC (Alicia Fry, Jerome Tokars, Marc-Alain Widdowson, Melissa Rolfes, Aaron Curns) and Nicaragua (Karena Vega, Eveling Aguilar, Jasmina Ampie, Vilmaricia Cerda, Yexica Chavez, Heydi Rodriguez, Wismar Whitman Ubau Andino, Noritza Martinez, Roger Lopez, Andrea Nuñez, Ubania Vargas, Jose Victor Zambrana, William Aviles, Raquel Burger-Calderon, Douglas Elizondo, Brenda Lopez).

***Financial support***. This work was supported by the Centers for Disease Control and Prevention through contract HHSD2002013M53890B within its Achieving Public Health Impact Through Research and task 200-2014-F-60406 to Abt Associates; and by the Biosciences Initiative at the University of Michigan through a Mid-career Biosciences Faculty Achievement Award to A. G.
