# Supplementary material for: Etiology of Acute Lower Respiratory Illness Hospitalizations Among Infants in 4 Countries
Source: Open Forum Infect Dis. 2023 Nov 16;10(12):ofad580. doi: 10.1093/ofid/ofad580 (PMC10733183; doi:10.1093/ofid/ofad580)
Supplement: ofad580_Supplementary_Data [file ofad580_supplementary_data.docx]

# Supplemental materials for “Etiology of acute lower respiratory illness hospitalizations among infants in four countries”

## Supplemental Methods

## Nested partially latent class model (NPLCM)

## The model assumes that the listed pathogens are detected by the assays with true or false positive rates depending on whether the pathogens are in the lung. False positive rates can be directly estimated from the control data; true positive rates are not fully statistically identified from data alone hence require informative priors.^1-3^ In a Bayesian framework, *baker* R package^4^ implements a posterior inference algorithm to estimate the fractions of cases caused by each of the causative pathogens along with the uncertainty quantification of the estimated fractions.

## Prior specification

To fit the Bayesian nested partially latent class models^1-3^ to the study data, we needed to specify priors for three sets of unknown parameters: the population etiologic fractions (PEF), the true positive rates (TPR) and the false positive rates (FPR) for each of the tested pathogens. For the population etiologic fractions, we used an overall Uniform prior (symmetric Dirichlet distribution with hyperparameters 1) so that no pathogen was favored. For the true positive rate (TPR) of each tested pathogen, we assigned independent Beta priors with the 2.5% and 97.5% quantiles representing the plausible lower (0.5) and upper (0.99) limits of the sensitivity of detecting the pathogen via RT-PCR of NP/OP swabs. The priors for TPRs are informative, meaning that it is not uniform over 0 and 1, which is the range of values of TPRs. The lower and upper limits are used to indicate that probability of the diagnostic test for a pathogen being positive given the true pathogen infecting the lung is more than a random guess *a priori*. We subsequently revised the TPR prior for RSV to have a lower bound of $0.75$ to improve model fit (as assessed from posterior predictive checks). For the FPRs (or, 1-specificity), because we can directly learn from the control data, we specify independent uniform priors between 0 and 1 and let the control data update them to values that are compatible with the study data.

## Supplemental Figure 1 – Upset plot of co-infection combinations

This figure shows the most observed co-infection combinations (across sites) in descending order. Participants were considered to be co-infected if PCR testing of combined NP/OP swabs resulted in multiple pathogen detections. The bar plot shows the number of participants observed to have a particular co-infection combination, while the matrix below displays which pathogens each co-infection combination refers to. Abbreviations: RSV, respiratory syncytial virus; HMPV, human metapneumovirus; RV/EV, rhinovirus/enterovirus; Cor43, coronavirus OC43; HPIV3, human parainfluenza virus 3.


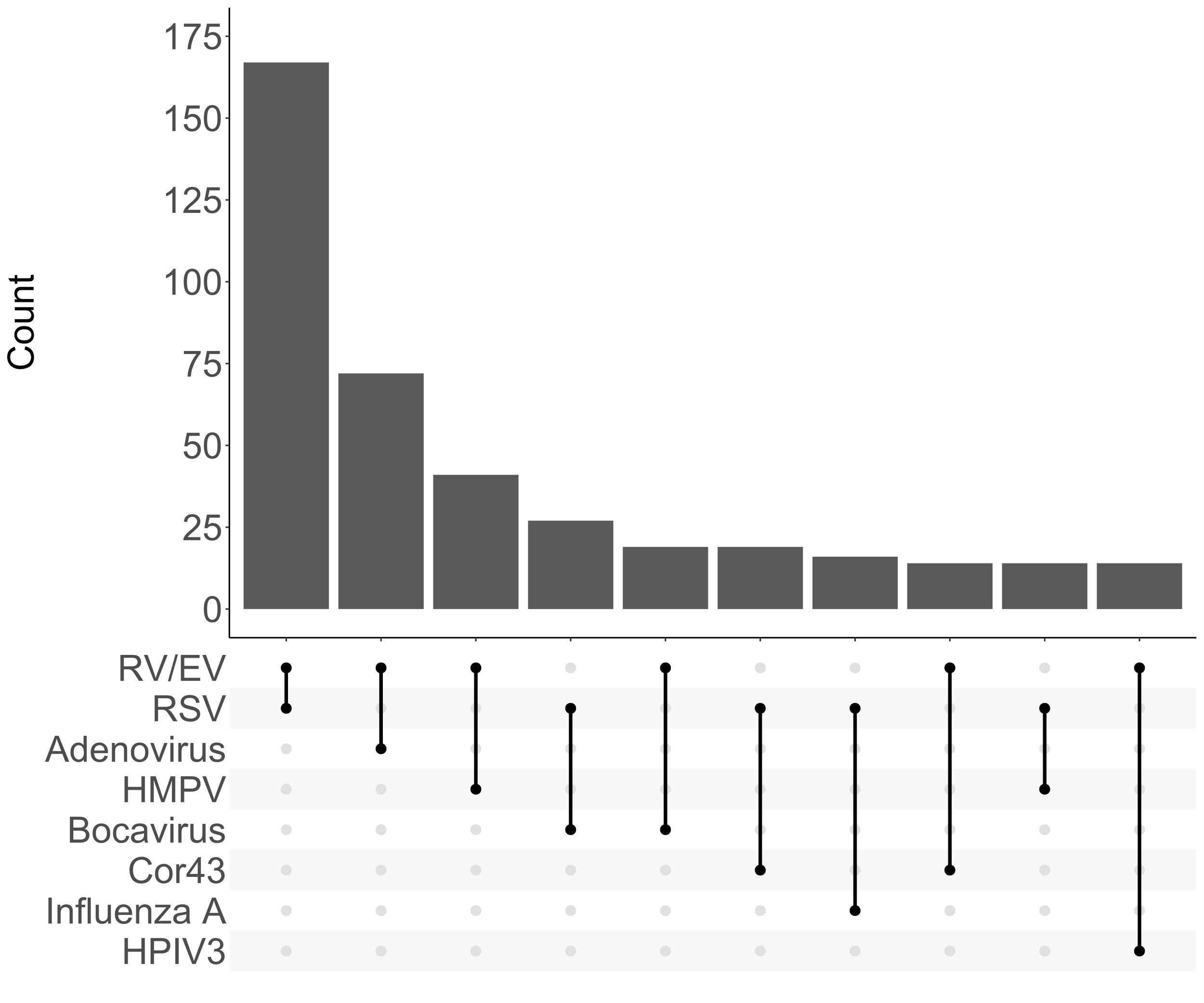


## Supplemental Figure 2 - Sensitivity analysis

As can be seen in Figure 1 of the manuscript, there were some periods in which the enrollment of non-ill controls was not well-matched to enrollment of hospitalized infants. Specifically, the beginning of the study period in Nicaragua and the middle of the study period in the Philippines appeared poorly matched. To assess the potential for this to bias our results, we excluded data from those time periods and reran the NPLCM. We have included the following figure showing the results of this sensitivity analysis. The population etiologic fractions observed in the sensitivity analysis (below) were largely consistent with those observed in the primary analysis (Supplemental Table 3).


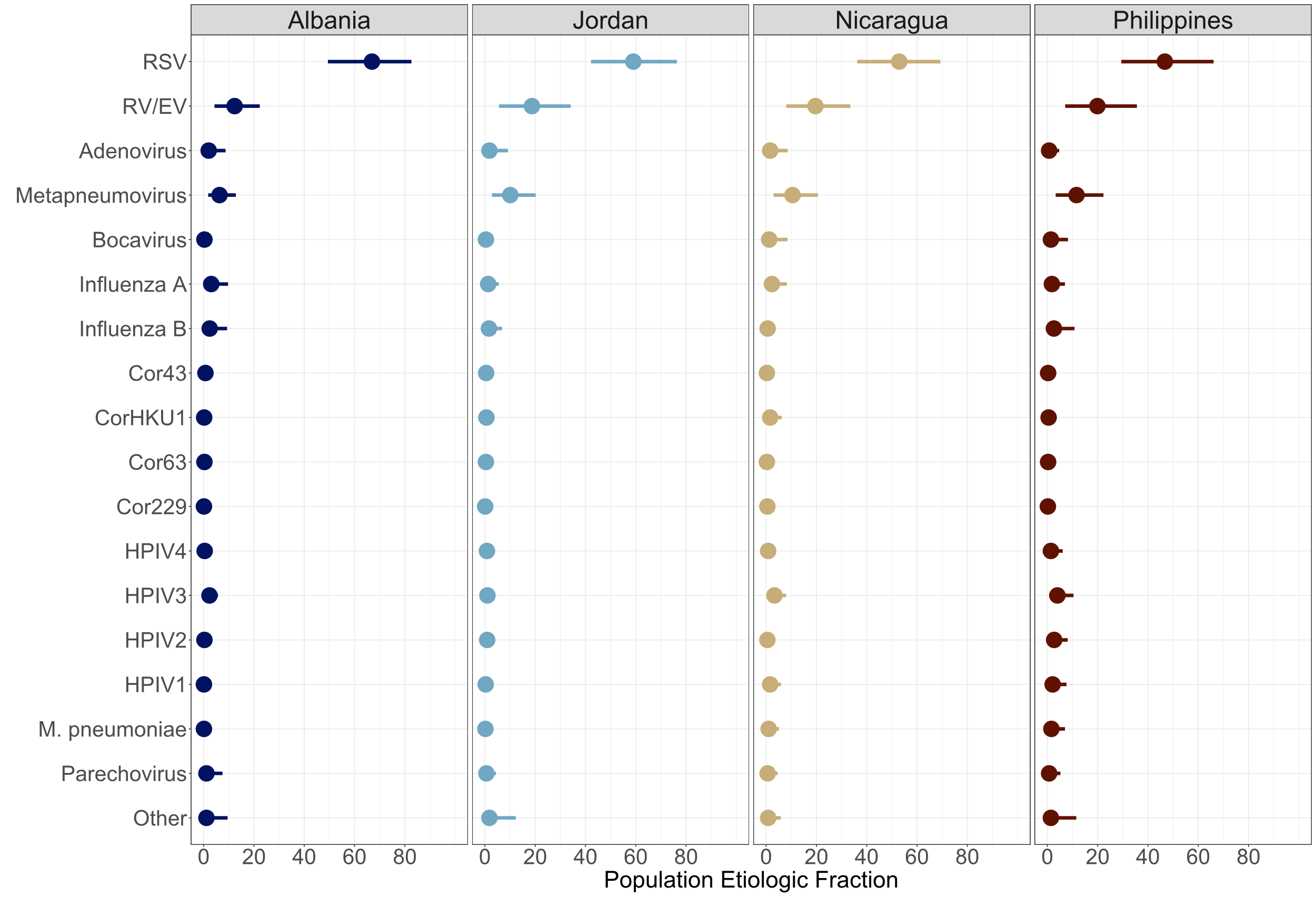


## Supplemental Figure 3 – PCR positives over time

This figure displays the number of PCR positives for each pathogen across sites by month. The number is scaled to be the proportion of the maximum number of positives over 1 month ($numberofPCRpositives/max(numberofPCRpositives$) per pathogen and study site. Time periods in white represent when samples were not being taken at a given site.


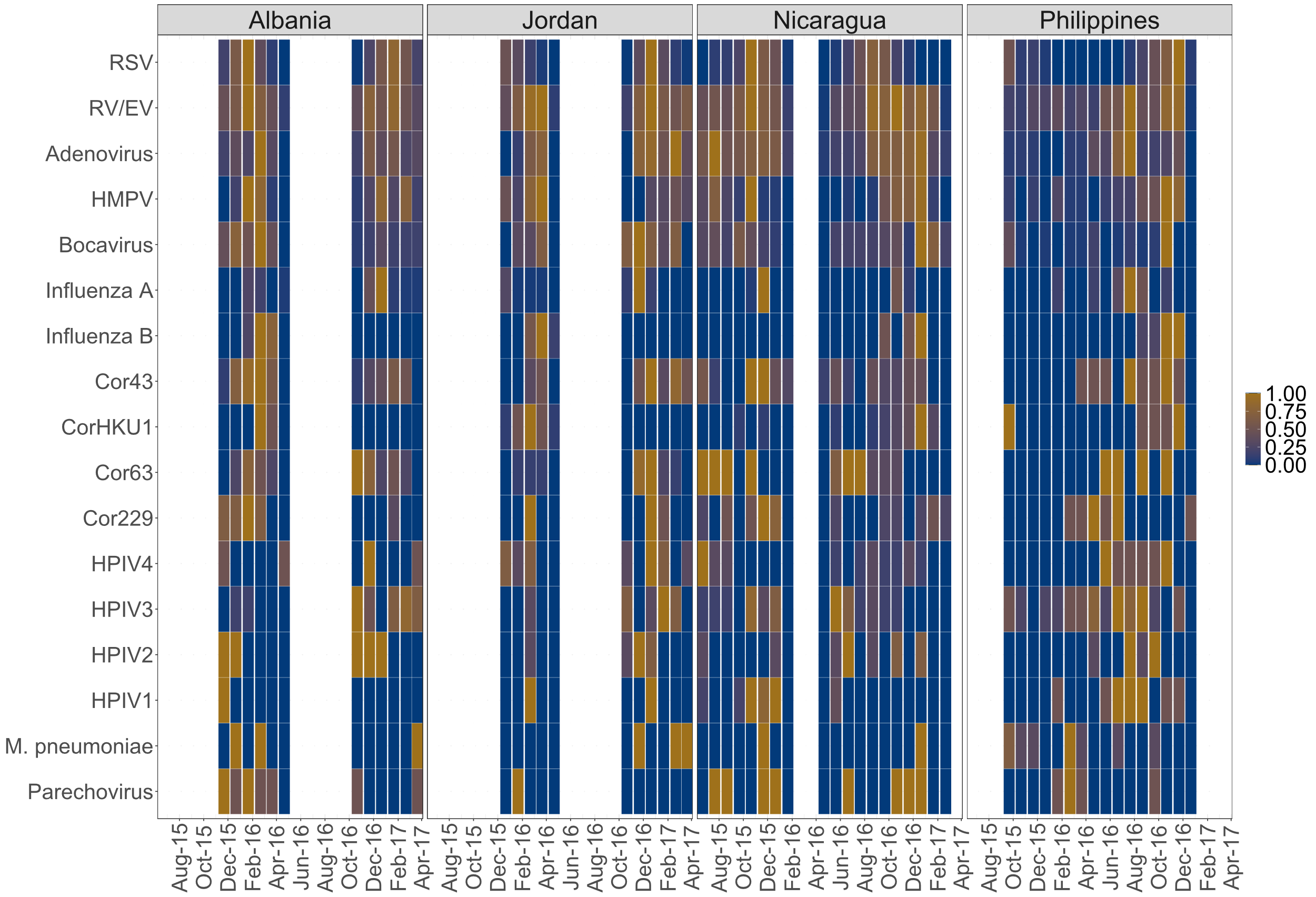


## Supplemental Figure 4 – Ratio of mean days hospitalized by infection type.

Panel A shows the mean number of days hospitalized for RSV, RV/EV single infections vs. RSV+RV/EV co-infections while panel B shows HAdV, RV/EV single infections vs. HAdV+RV/EV co-infections. Abbreviations: RSV, respiratory syncytial virus; RV/EV, rhinovirus/enterovirus; HAdV, human adenovirus.


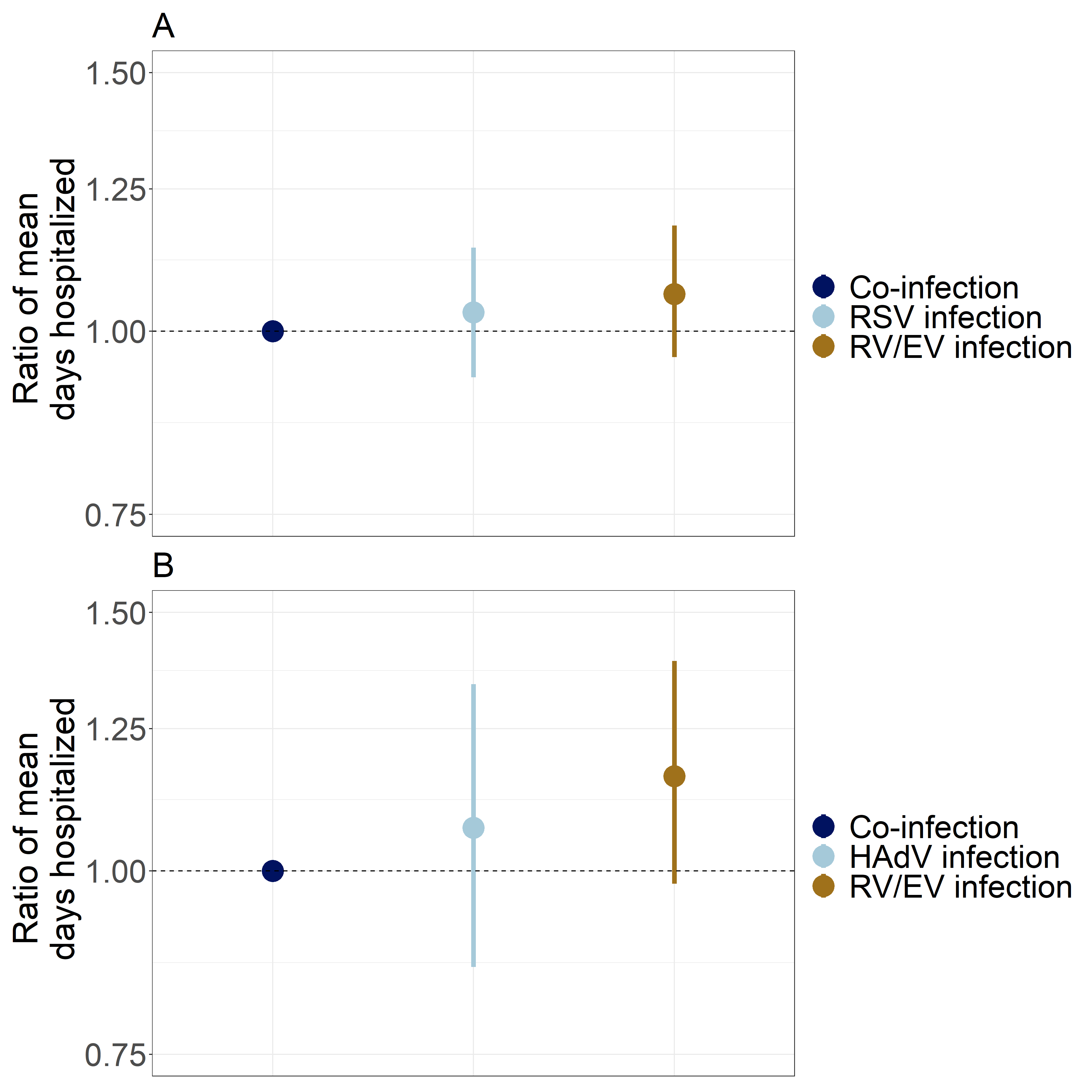


##

## Supplemental Table 1 – Prevalence by study site and cohort

Prevalence estimates represent the proportion of PCR+ (from combined NP/OP swabs)/total number in the specified group. Abbreviations: RSV, respiratory syncytial virus; HMPV, human metapneumovirus; RV/EV, rhinovirus/enterovirus; Cor43, coronavirus OC43; CorHKU1, coronavirus HKU1; Cor63, coronavirus NL63; Cor229, coronavirus 229E; HPIV1-HPIV4, human parainfluenza virus 1-4.

|  | **Albania** | | **Jordan** | | **Nicaragua** | | **Philippines** | |
| --- | --- | --- | --- | --- | --- | --- | --- | --- |
| **Characteristic** | **Hospitalized**,  N = 1,032^1^ | **Non-ill**,  N = 363^1^ | **Hospitalized**,  N = 1,056^1^ | **Non-ill**,  N = 173^1^ | **Hospitalized**,  N = 936^1^ | **Non-ill**,  N = 399^1^ | **Hospitalized**,  N = 607^1^ | **Non-ill**,  N = 133^1^ |
| Parechovirus | 7 (0.7%) | 2 (0.6%) | 0 (0%) | 1 (0.6%) | 8 (0.9%) | 0 (0%) | 5 (0.8%) | 0 (0%) |
| M. pneumoniae | 3 (0.3%) | 0 (0%) | 2 (0.2%) | 1 (0.6%) | 4 (0.4%) | 0 (0%) | 10 (1.6%) | 0 (0%) |
| HPIV1 | 1 (<0.1%) | 0 (0%) | 1 (<0.1%) | 1 (0.6%) | 15 (1.6%) | 3 (0.8%) | 10 (1.6%) | 0 (0%) |
| HPIV2 | 4 (0.4%) | 1 (0.3%) | 7 (0.7%) | 0 (0%) | 11 (1.2%) | 0 (0%) | 8 (1.3%) | 0 (0%) |
| HPIV3 | 22 (2.1%) | 2 (0.6%) | 9 (0.9%) | 0 (0%) | 24 (2.6%) | 5 (1.3%) | 26 (4.3%) | 1 (0.8%) |
| HPIV4 | 5 (0.5%) | 0 (0%) | 10 (0.9%) | 2 (1.2%) | 18 (1.9%) | 1 (0.3%) | 8 (1.3%) | 0 (0%) |
| Cor229 | 5 (0.5%) | 5 (1.4%) | 3 (0.3%) | 2 (1.2%) | 15 (1.6%) | 4 (1.0%) | 6 (1.0%) | 2 (1.5%) |
| Cor63 | 13 (1.3%) | 5 (1.4%) | 15 (1.4%) | 6 (3.5%) | 18 (1.9%) | 5 (1.3%) | 3 (0.5%) | 1 (0.8%) |
| CorHKU1 | 5 (0.5%) | 1 (0.3%) | 19 (1.8%) | 3 (1.7%) | 21 (2.2%) | 5 (1.3%) | 6 (1.0%) | 1 (0.8%) |
| Cor43 | 39 (3.8%) | 13 (3.6%) | 24 (2.3%) | 0 (0%) | 29 (3.1%) | 13 (3.3%) | 10 (1.6%) | 0 (0%) |
| Influenza B | 24 (2.3%) | 0 (0%) | 10 (0.9%) | 2 (1.2%) | 3 (0.3%) | 1 (0.3%) | 10 (1.6%) | 0 (0%) |
| Influenza A | 43 (4.2%) | 5 (1.4%) | 48 (4.5%) | 9 (5.2%) | 25 (2.7%) | 4 (1.0%) | 13 (2.1%) | 1 (0.8%) |
| Bocavirus | 41 (4.0%) | 9 (2.5%) | 10 (0.9%) | 4 (2.3%) | 48 (5.1%) | 9 (2.3%) | 44 (7.2%) | 3 (2.3%) |
| HMPV | 43 (4.2%) | 6 (1.7%) | 48 (4.5%) | 2 (1.2%) | 77 (8.2%) | 12 (3.0%) | 49 (8.1%) | 10 (7.5%) |
| Adenovirus | 59 (5.7%) | 12 (3.3%) | 37 (3.5%) | 3 (1.7%) | 114 (12%) | 41 (10%) | 41 (6.8%) | 2 (1.5%) |
| RV/EV | 250 (24%) | 107 (29%) | 226 (21%) | 48 (28%) | 361 (39%) | 133 (33%) | 179 (29%) | 57 (43%) |
| RSV | 438 (42%) | 32 (8.8%) | 358 (34%) | 19 (11%) | 208 (22%) | 10 (2.5%) | 123 (20%) | 9 (6.8%) |
| ^1^n (%) | | | | | | | | |

## Supplemental Table 2 – Prevalence by age and cohort

Prevalence estimates represent the proportion of PCR+ (from combined NP/OP swabs)/total number in the specified group. Abbreviations: RSV, respiratory syncytial virus; HMPV, human metapneumovirus; RV/EV, rhinovirus/enterovirus; Cor43, coronavirus OC43; CorHKU1, coronavirus HKU1; Cor63, coronavirus NL63; Cor229, coronavirus 229E; HPIV1-HPIV4, human parainfluenza virus 1-4.

|  | **<14 weeks** | | **14-26 weeks** | | **27-39 weeks** | | **40-52 weeks** | |
| --- | --- | --- | --- | --- | --- | --- | --- | --- |
| **Characteristic** | **Hospitalized**,  N = 1,793^1^ | **Non-ill**,  N = 431^1^ | **Hospitalized**,  N = 706^1^ | **Non-ill**,  N = 247^1^ | **Hospitalized**,  N = 599^1^ | **Non-ill**,  N = 181^1^ | **Hospitalized**,  N = 533^1^ | **Non-ill**,  N = 209^1^ |
| Parechovirus | 3 (0.2%) | 0 (0%) | 8 (1.1%) | 0 (0%) | 4 (0.7%) | 0 (0%) | 5 (0.9%) | 3 (1.4%) |
| M. pneumoniae | 4 (0.2%) | 1 (0.2%) | 7 (1.0%) | 0 (0%) | 6 (1.0%) | 0 (0%) | 2 (0.4%) | 0 (0%) |
| HPIV1 | 5 (0.3%) | 2 (0.5%) | 9 (1.3%) | 1 (0.4%) | 7 (1.2%) | 1 (0.6%) | 6 (1.1%) | 0 (0%) |
| HPIV2 | 6 (0.3%) | 1 (0.2%) | 7 (1.0%) | 0 (0%) | 6 (1.0%) | 0 (0%) | 11 (2.1%) | 0 (0%) |
| HPIV3 | 30 (1.7%) | 4 (0.9%) | 20 (2.8%) | 1 (0.4%) | 19 (3.2%) | 1 (0.6%) | 12 (2.3%) | 2 (1.0%) |
| HPIV4 | 17 (0.9%) | 0 (0%) | 11 (1.6%) | 1 (0.4%) | 9 (1.5%) | 0 (0%) | 4 (0.8%) | 2 (1.0%) |
| Cor229 | 11 (0.6%) | 5 (1.2%) | 8 (1.1%) | 4 (1.6%) | 5 (0.8%) | 0 (0%) | 5 (0.9%) | 4 (1.9%) |
| Cor63 | 22 (1.2%) | 7 (1.6%) | 11 (1.6%) | 5 (2.0%) | 6 (1.0%) | 3 (1.7%) | 10 (1.9%) | 2 (1.0%) |
| CorHKU1 | 24 (1.3%) | 4 (0.9%) | 6 (0.8%) | 2 (0.8%) | 13 (2.2%) | 0 (0%) | 8 (1.5%) | 4 (1.9%) |
| Cor43 | 35 (2.0%) | 9 (2.1%) | 23 (3.3%) | 10 (4.0%) | 27 (4.5%) | 5 (2.8%) | 17 (3.2%) | 2 (1.0%) |
| Influenza B | 8 (0.4%) | 0 (0%) | 9 (1.3%) | 1 (0.4%) | 19 (3.2%) | 0 (0%) | 11 (2.1%) | 2 (1.0%) |
| Influenza A | 48 (2.7%) | 6 (1.4%) | 28 (4.0%) | 5 (2.0%) | 28 (4.7%) | 4 (2.2%) | 25 (4.7%) | 4 (1.9%) |
| Bocavirus | 27 (1.5%) | 4 (0.9%) | 27 (3.8%) | 8 (3.2%) | 46 (7.7%) | 7 (3.9%) | 43 (8.1%) | 6 (2.9%) |
| HMPV | 57 (3.2%) | 13 (3.0%) | 57 (8.1%) | 4 (1.6%) | 53 (8.8%) | 5 (2.8%) | 50 (9.4%) | 8 (3.8%) |
| Adenovirus | 47 (2.6%) | 18 (4.2%) | 49 (6.9%) | 12 (4.9%) | 78 (13%) | 10 (5.5%) | 77 (14%) | 18 (8.6%) |
| RV/EV | 444 (25%) | 118 (27%) | 232 (33%) | 102 (41%) | 183 (31%) | 68 (38%) | 157 (29%) | 57 (27%) |
| RSV | 606 (34%) | 26 (6.0%) | 234 (33%) | 18 (7.3%) | 166 (28%) | 11 (6.1%) | 121 (23%) | 15 (7.2%) |
| ^1^n (%) | | | | | | | | |

##

## Supplemental Table 3 – Symptom development among non-ill controls

A subset of the controls in year 2 were followed after enrollment to determine whether they would develop symptoms. This table shows the RT-PCR results for those controls stratified by whether they did develop symptoms. Abbreviations: RSV, respiratory syncytial virus; HMPV, human metapneumovirus; RV/EV, rhinovirus/enterovirus; Cor43, coronavirus OC43; CorHKU1, coronavirus HKU1; Cor63, coronavirus NL63; Cor229, coronavirus 229E; HPIV1-HPIV4, human parainfluenza virus 1-4.

| **Characteristic** | **Developed symptoms**, N = 92^1^ | **Did not develop symptoms**, N = 434^1^ |
| --- | --- | --- |
| Parechovirus | 0 (0%) | 0 (0%) |
| M. pneumoniae | 0 (0%) | 1 (0.2%) |
| HPIV1 | 0 (0%) | 0 (0%) |
| HPIV2 | 1 (1.1%) | 0 (0%) |
| HPIV3 | 0 (0%) | 4 (0.9%) |
| HPIV4 | 2 (2.2%) | 1 (0.2%) |
| Cor229 | 1 (1.1%) | 3 (0.7%) |
| Cor63 | 2 (2.2%) | 10 (2.3%) |
| CorHKU1 | 2 (2.2%) | 4 (0.9%) |
| Cor43 | 0 (0%) | 6 (1.4%) |
| Influenza B | 1 (1.1%) | 0 (0%) |
| Influenza A | 3 (3.3%) | 11 (2.5%) |
| Bocavirus | 0 (0%) | 11 (2.5%) |
| HMPV | 4 (4.3%) | 10 (2.3%) |
| Adenovirus | 6 (6.5%) | 23 (5.3%) |
| RV/EV | 35 (38%) | 146 (34%) |
| RSV | 15 (16%) | 22 (5.1%) |
| ^1^n (%) | | |

## Supplemental Table 4 – PEF values for all pathogens by study site

This table displays the population etiologic fraction (PEF) for each pathogen with 95% credible intervals (CrI) by site (marginalized over age and sex). Abbreviations: RSV, respiratory syncytial virus; HMPV, human metapneumovirus; RV/EV, rhinovirus/enterovirus; Cor43, coronavirus OC43; CorHKU1, coronavirus HKU1; Cor63, coronavirus NL63; Cor229, coronavirus 229E; HPIV1-HPIV4, human parainfluenza virus 1-4.

|  | Albania | Jordan | Nicaragua | Philippines |
| --- | --- | --- | --- | --- |
|  | PEF (95% CrI)^a^ | PEF (95% CrI)^a^ | PEF (95% CrI)^a^ | PEF (95% CrI)^a^ |
| Influenza A | 3.3 (0.2,9.9) | 1.4 (0,5.4) | 1.8 (0.1,6.3) | 2.2 (0.1,7.2) |
| Influenza B | 2.4 (0.1,8.9) | 1.5 (0.1,6.7) | 0.4 (0,2.3) | 2 (0.1,7.3) |
| RSV | 65.2 (46.3,79.6) | 57.1 (40.2,71.8) | 44.9 (31.5,59.5) | 34.9 (20,49) |
| Cor63 | 0.2 (0,1.6) | 0.4 (0,2.7) | 0.5 (0,2.8) | 0.2 (0,1.7) |
| Cor229 | 0.1 (0,0.9) | 0.1 (0,1.1) | 0.5 (0,3.3) | 0.2 (0,1.7) |
| Cor43 | 0.7 (0,3.9) | 0.5 (0,3.3) | 0.2 (0,1.6) | 0.7 (0,3.8) |
| CorHKU1 | 0.2 (0,1.2) | 0.6 (0,3.2) | 1 (0,4.4) | 0.4 (0,2.3) |
| HPIV2 | 0.3 (0,1.3) | 1 (0.1,3.8) | 0.4 (0,1.9) | 2.6 (0.3,7.6) |
| HPIV3 | 2.1 (0.6,4.9) | 0.9 (0,2.9) | 2.8 (0.6,6.7) | 6.7 (2.3,14) |
| HPIV4 | 0.4 (0,1.8) | 0.8 (0,3.4) | 1.4 (0,5.1) | 1.1 (0,4.4) |
| HPIV1 | 0.1 (0,0.8) | 0.3 (0,1.7) | 1.2 (0,4.5) | 2.2 (0.1,7.1) |
| Metapneumovirus | 6.4 (1.7,12.8) | 10.1 (2.7,20) | 12 (3.2,22.7) | 10.3 (2.6,20.5) |
| Bocavirus | 0.2 (0,1.6) | 0.3 (0,2.4) | 0.7 (0,5) | 1.8 (0,8.8) |
| M. pneumoniae | 0.1 (0,0.8) | 0.2 (0,1.3) | 0.8 (0,4) | 1.6 (0,6.8) |
| Adenovirus | 1.6 (0,6.8) | 1.5 (0,8.9) | 2.8 (0,11.3) | 1.2 (0,6.4) |
| RV/EV | 13.4 (4.3,25.4) | 20.5 (8,35.4) | 27.1 (12.1,45.4) | 25.6 (10.2,45.5) |
| Parechovirus | 1.4 (0,11.5) | 0.4 (0,3.4) | 0.4 (0,3.5) | 2.7 (0,19.2) |
| Other | 1.7 (0,13.8) | 2.2 (0,15.6) | 1 (0,7.5) | 3.6 (0,22.1) |
| ^a^Population Etiologic Fraction (95% Credible Interval) | | | | |

References

1. Deloria Knoll M, Fu W, Shi Q, et al. Bayesian Estimation of Pneumonia Etiology: Epidemiologic Considerations and Applications to the Pneumonia Etiology Research for Child Health Study. Clin Infect Dis 2017;64(suppl_3):S213-s227. (In eng). DOI: 10.1093/cid/cix144.

2. Wu Z, Deloria-Knoll M, Hammitt LL, Zeger SL. Partially latent class models for case-control studies of childhood pneumonia aetiology. J R Stat Soc Ser C Appl Stat 2016;65(1):97-114. (In eng). DOI: 10.1111/rssc.12101.

3. Wu Z, Deloria-Knoll M, Zeger SL. Nested partially latent class models for dependent binary data; estimating disease etiology. Biostatistics 2017;18(2):200-213. (In eng). DOI: 10.1093/biostatistics/kxw037.

4. Chen IB, Shi Q, Zeger SL, Wu Z. baker: An R package for Nested Partially-Latent Class Models. arXiv preprint arXiv:220211778 2022.
